# Gender and age-related variations in rumen fermentation and microbiota of Qinchuan cattle

**DOI:** 10.5713/ab.24.0328

**Published:** 2024-10-24

**Authors:** Yueting Pan, Huaxuan Li, Juze Wang, Xiaolei Sun, Entang Liang, Juntao Guo, Jianfang Wang, Ke Zhang, Bingzhi Li, Mengqi Zan, Wucai Yang, Linsen Zan

**Affiliations:** 1 College of Animal Science and Technology, Northwest A&F University, Yangling, China; 2 National Beef Cattle Improvement Center, Yangling, China; 3 Key Laboratory for Efficient Ruminant Breeding Technology of Higher Education Institutions in Shaanxi Province, Yangling Vocational & Technical College, Yangling, China

**Keywords:** Age-related Variations, Gender Differences, Qinchuan Cattle, Rumen Microbiota, Volatile Fatty Acid (VFA)

## Abstract

**Objective:**

Our study aimed to investigate the gender and age-related variations in rumen fermentation, serum metabolites, and microbiota in Qinchuan cattle.

**Methods:**

A total of 38 Qinchuan beef cattle were selected and maintained on a uniform diet for three months. Rumen fluid and blood samples were collected to determine rumen fermentation, serum metabolites, and microbial 16S rRNA sequencing.

**Results:**

The results revealed that the concentration of rumen butyrate in female Qinchuan cattle was significantly higher than in males (p<0.05). Isobutyrate, butyrate, and isovalerate exhibited significant age-related differences. Females exhibited lower serum glucose (GLU) and higher triglycerides (TG), nonesterifiedfatty acid (NEFA) levels compared to males (p<0.05). Serum albumin (ALB) and urea (UA) levels increased with age (p<0.05). Furthermore, the alpha diversity of rumen bacteria improved with age (p<0.05), with no gender differences observed. Males had higher relative abundances of Bacteroidota, Verrucomicrobiota, and Cyanobacteria, while females had higher Firmicutes and Desulfobacterota (p<0.05). The cellulose-degrading genus *Ruminococcus* and propionate-producing genus *Succiniclasticum* were more abundant in females, whereas the anti-inflammatory genus *Lachnospiraceae_NK4A136_group* and the hemicellulose-degrading genus *Prevotella* were more abundant in males (p<0.05). Age-related differences in bacteria were found in *Pseudobutyrivibrio* and several members of the Lachnospiraceae. Functional prediction indicated that “Amino acid metabolism” and “Lipid metabolism” were mainly enriched in females, whereas “Carbohydrate metabolism” and “Glycan biosynthesis and metabolism” were enriched in males (p<0.05). RDA analysis highlighted butyrate as a key factor influencing the rumen bacterial community. *NK4A214_group* and *Ruminococcus* were positively correlated with butyrate, while *Prevotella* and *Pseudobutyrivibrio* were negatively correlated with butyrate (p<0.05).

**Conclusion:**

We observed a significant improvement in the diversity and stability of rumen microbiota as age increased. *Ruminococcus*, *NK4A214_group*, and *Prevotella* were likely contributors to variations in energy utilization and fat deposition between male and female Qinchuan cattle.

## INTRODUCTION

Ruminants rely on a diverse and robust rumen microbiota for the efficient digestion of plant fibers, which are converted into volatile fatty acids (VFAs), microbial proteins, ammonia and other metabolites essential for growth [1]. The rumen contains a diverse array of microorganisms, such as bacteria, protozoa, and fungi, which co-evolve with the host to influence the phenotype and carry out vital functions in metabolism, digestion, and immunity [2]. Diet, environment, and host genetics were the primary drivers of the observed variations in the rumen microbiota community structure. Animal performance traits, such as average daily gain and feed efficiency have also been associated with specific microbial states in the rumen, indicating that microbiota can influence livestock productivity [3].

Gender and age, fundamental characteristics of individual organisms, significantly influence the composition of the gut microbiota community [4]. Gender differences may lead to variations in hormone levels, dietary preferences, and metabolic functions, thereby impacting the structure of the gut microbiota [5]. Studies have indicated that females tend to have higher intestinal microbial diversity and a greater abundance of antibiotic-resistance genes than males [6]. Additionally, host phenotypes, such as body mass index (BMI) and diastolic blood pressure, inversely correlate with the core microbiota, which is influenced by gender [7]. Notably, research conducted on non-obese diabetic (NOD) mouse models revealed that transplanting male colonic contents profoundly reshaped the gut microbiota of female NOD mice. This intervention alleviated pancreatic inflammation and reduced insulin antibody production [8], suggesting a gender-specific role of the gut microbiota in modulating the occurrence of host diseases. Furthermore, age significantly influences the dynamic assembly of the gut microbiota. Changes in rumen structure and physiological characteristics correlate with the development of the rumen microbiota [9]. Studies have demonstrated significant changes in the composition of rumen microbiota between 1-day-old and 3-day-old calves, despite their identical diets and nearly identical symptoms [10]. Following calf birth, aerobic bacteria in the rumen rapidly deplete the available oxygen, prompting a shift from aerobic to anaerobic conditions in the microbial ecological environment. It has been found that age-related microbial changes are closely related to host inflammation [11]. Consequently, changes in ecological niches lead to a restructuring of the microbial community by the rumen microbiota. The initial bacterial communities colonizing the rumen differ significantly from those found in mature animals. Increasing age contributes to enhanced complexity and stability within these communities [10]. Additionally, females exhibited higher carcass fat and back fat content compared to males, and intramuscular fat content significantly increased with age [12]. Studies have indicated that tail fat deposition was negatively correlated with the abundance of Verrucomicrobiota and *Bacteroides* in the colon of Small-Tailed Han sheep [13], while the abundance of *Moryella* in the rumen was positively correlated with intramuscular fat content in Angus× Simmental steers [14]. These studies have demonstrated that the gut microbiota is influenced by the gender and age of the host, and is closely associated with host phenotypes such as fat deposition. However, our understanding of the gender and age disparities in the composition and functionality of rumen microbiota among ruminants is still limited due to various constraints, such as animal breeds, feeding management practices, geographical locations, and environmental conditions.

Therefore, we conducted a comparative analysis of Qinchuan cattle with uniform genetic backgrounds and consistent feeding management practices, varying only in gender and age, to explore their respective impacts on ruminal fermentation function and microbial community structure. Our study lays the groundwork for improving ruminant production efficiency by strategically regulating rumen microbiota. It also offers valuable insights into conserving and strategically utilizing genetic resources in Chinese local yellow cattle.

## MATERIALS AND METHODS

### Animal care

The management and handling of experimental animals are carried out in accordance with the Animal Welfare and Ethical Rules of the Laboratory Animal Management Committee of Northwest A&F University of Science and Technology in China and have been approved (protocol number: NWAFUCAST2018-168).

### Experiment design and sample collection

The study was conducted at the Qinchuan Cattle Breeding Farm of the National Beef Cattle Improvement Center. A total of 38 QinChuan beef cattle, aged 3 months (6 males and 6 females), 9 months (7 males and 7 females), and 15 months (6 males and 6 females), with good health and consistent feeding practices, were selected. The experimental animals, grouped by gender and age, were all maintained on a same diet for three months. The diet consisted of corn silage, wheat straw, and concentrate, fed twice daily at 6:00 AM and 4:00 PM. The feed was manually mixed according to the formulation and provided to the experimental animals. The feeding amount was set to 110% of the previous intake to ensure feed remained each day. The concentrate was formulated according to the Beef Cattle Feeding Standard (NY/T 81-2004). The composition and nutrient levels of the diet were detailed in Table 1. Clean and ample drinking water was provided, and standard immunization procedures were followed.

Rumen fluid and blood samples were collected when the cattle were 6 months old (QC_6_), 12 months old (QC_12_), and 18 months old (QC_18_). Rumen fluid was collected two hours after the morning feeding using an oral rumen tube, then filtered through gauze, and divided into 5 mL centrifuge tubes. Samples were immediately frozen in liquid nitrogen and stored at −80°C for subsequent determination of rumen fermentation performance and microbial 16S rRNA sequencing. Blood samples were obtained from the jugular vein of cattle on an empty stomach using conventional vacutainer tubes. The samples were then incubated at 25°C for 4–6 h, and the serum was subsequently separated by centrifugation at 3,000×g for 15 min. Serum samples, each 500 μL, were divided into 1.5 mL Eppendorf tubes and stored at −80°C for the analysis of serum metabolites.

### Chemical analysis

The chemical composition of the feed sample was determined after drying at 65°C for 72 h and crushing it through a 1 mm screen. Nitrogen content was determined using the Kjeldahl method with copper sulphate and potassium sulphate (1:10, w/w) as a catalyst (UDK159, VELP, Usmate, MB, Italy). Crude protein (CP) content was calculated as N×6.25. The content of neutral detergent fiber (NDF) and acid detergent fiber (ADF) was analyzed using a fibre analyser (F800, Hanon, Jinan, China), following the method described by Van Soest et al [15]. Ash content was measured using a muffle furnace at 550°C for 5 h, with preliminary ashing in an electric heating panel (F47910-33, Thermo Scientific, Waltham, MA, USA). Organic matter (OM) content was calculated by subtracting the ash content from 100%. The ether extract (EE) content was determined using an automatic Soxhlet extractor (SOX606, Hanon).

The concentration of VFAs in the rumen fluid was determined using gas chromatography-flame ionization detection (GC-FID) (internal standard method, GC-6850, Agilent, Santa Clara, CA, USA), with crotonic acid as serving as the internal standard. Ammonia nitrogen (NH_3_-N) concentration was measured by using an Ultraviolet-visible spectrophotometer (colorimetric method; Cary 60, Agilent). The concentration of serum metabolites was assessed using an automatic chemistry analyzer (BK-400, Biobase, Jinan, China).

### DNA extraction and high-throughput sequencing

The TIANamp Stool DNA Kit (Tiangen Biochemical Technology Co., Ltd., Beijing, China) was used to extract the total DNA of bacterial in rumen fluid. The V3–V4 region of the 16S rRNA genes in the total DNA was amplified using specific primers (F: ACTCCTACGGGAGGCAGCA; R: GGACTACHVGGGTWTCTAAT), with a sequencing adapter added to the end of the primer before PCR amplification. PCR products were then purified, quantified, and normalized to construct a sequencing library. Following quality assessment, the amplified products were sequenced on the Illumina NovaSeq 6000 platform (Illumina, San Diego, CA, USA), with the sequencing procedures and raw data quality control entrusted to Biomic Biotechnology Co., Ltd. (Beijing, China).

### Bioinformatics analysis

After filtering, removing primer sequences and chimeras, the DADA2 algorithm [16] within QIIME2 2020.6 [17] was used to denoise the data, applying a threshold of 0.005% to filter amplicon sequence variants (ASVs). The SILVA database (Release138, http://www.arb-silva.de) served as the reference database, and the Naïve Bayes classifier annotated feature sequences, classified species, and filtered features with less than 2% sequence abundance. Alpha diversity (Chao1 and Shannon index) and beta diversity were assessed using QIIME2 2020.6 [17]. Principal Coordinates Analysis (PCoA) based on Bray-Curtis dissimilarity visualized differences in the bacterial community among groups, followed by permutational multivariate analysis of variance (PERMANOVA) to test the significance of these differences. Gender and age differences in rumen microorganisms at the phylum and genus levels were analyzed using STAMP (v2.1.3) [18] via Welch’s t-test, with unclassified taxa and relative abundances less than 0.1% being filtered out. Furthermore, functions from the Kyoto Encyclopedia of Genes and Genomes (KEGG) were predicted across different genders and ages using PICRUSt2 (Phylogenetic Investigation of Communities by Reconstruction of Unobserved States) [19]. Redundancy analysis (RDA) in CANOCO5 was utilized to examine the correlations among serum metabolites, rumen ammonia and VFAs production and dominant bacteria in the rumen.

### Statistics and Analysis

To investigate the changes in serum metabolites, rumen ammonia, and VFAs production variations in gender and age, a general linear model (GLM) procedure in SPSS 26.0 was employed. The analysis considered the fixed effects of gender and age, as well as the interaction between gender and age. The Pearson correlation coefficients (*r*) were also calculated using SPSS 26.0. Data were expressed as mean±standard error. Differences between groups were considered significant at p<0.05, while a p-value between 0.05 and 0.1 indicated a significant trend.

## RESULTS

### Rumen fermentation performance

There were no significant differences in the concentration of rumen NH_3_-N between genders and ages (p>0.05). However, the concentration of butyrate in the rumen of females was significantly higher, showing a 34.8% increase compared to males (p = 0.023). Nevertheless, no significant differences were observed in the concentrations of total VFAs, acetate, propionate, and other VFAs between males and females (p>0.05). Furthermore, compared to QC_6_, the concentrations of isobutyrate, butyrate, and isovalerate in the rumen of QC_18_ were elevated by 45.3%, 50.9%, and 49.6% (p<0.05), respectively. Additionally, the concentrations of acetate and total VFAs in the rumen significantly increased with age (p = 0.081 and p = 0.089, respectively). However, the interaction between gender and age had no significant effects on NH_3_-N and VFAs (p>0.05, Table 2).

### Serum metabolites

Analysis of serum metabolites in Qinchuan cattle of different genders showed that the content of cholinesterase (CHE) and glucose (GLU) in the females was significantly lower than in males (p<0.05). Conversely, the levels of total cholesterol (CHO) and triglycerides (TG) were significantly higher in females than in males. Moreover, the concentration of α-hydroxybutyrate dehydrogenase (α-HBDH) in females exhibited a rising trend compared to males (p = 0.062). Furthermore, the albumin (ALB) and urea (UA) contents in QC_6_ was significantly higher compared to QC_12_ and QC_18_ (p<0.01). Interestingly, the total bile acid (TBA) content gradually decreased with age (p = 0.059), while the α-HBDH content tended to increase (p = 0.055, Table 3).

### Microbial diversity

**Figure 1:**
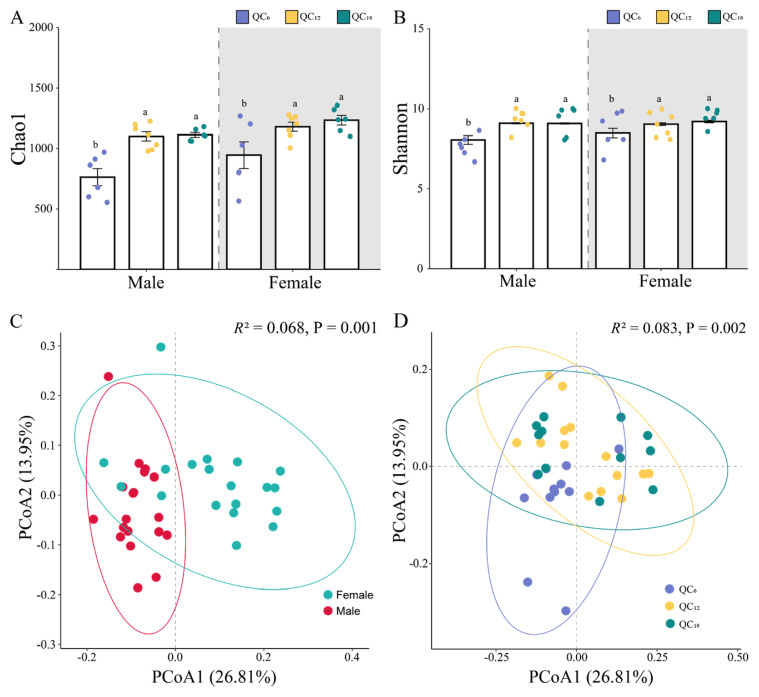
Gender and age differences in rumen microbial diversity in Qinchuan cattle. The histogram display variations in rumen microbial α-diversity by gender and age as measured by the Chao1 index (A) and Shannon index (B). β-diversity, based on Bray-Curtis PCoA, illustrated the differences in microbial community composition by gender (C) and age (D). ^a,b^ Different lowercase letters indicated significant differences (p<0.05), while identical lowercase letters denote no significant differences (p>0.05). PCoA, principal coordinates analysis.

A total of 22,443 ASVs were obtained from 38 samples using 16S rRNA sequencing. The rarefaction curves for all samples have nearly reached a plateau, indicating that the number of sequencing samples and the read coverage for each sample were sufficient to capture the diversity (Supplement 1). Alpha diversity of the rumen microbial communities was assessed using the Chao1 and Shannon indices. No significant differences were observed in the Chao1 and Shannon indices between males and females (p>0.05, Figures 1A, 1B). However, the Chao1 and Shannon indices for the QC_12_ and QC_18_ groups were significantly higher than those for the QC_6_ group, indicating an increase in microbial diversity and evenness with age (p<0.05). PCoA based on Bray-Curtis distance at ASV-level and the PERMANOVA revealed the differences in microflora structure between male and female group (*R*^2^ = 0.068, p = 0.001) and among the age groups (*R*^2^ = 0.083, p = 0.002). This suggests the higher spatial heterogeneity and lower community similarity in the rumen microbial communities among different genders and ages of Qinchuan cattle (Figures 1C, 1D).

### Microbial composition

**Figure 2:**
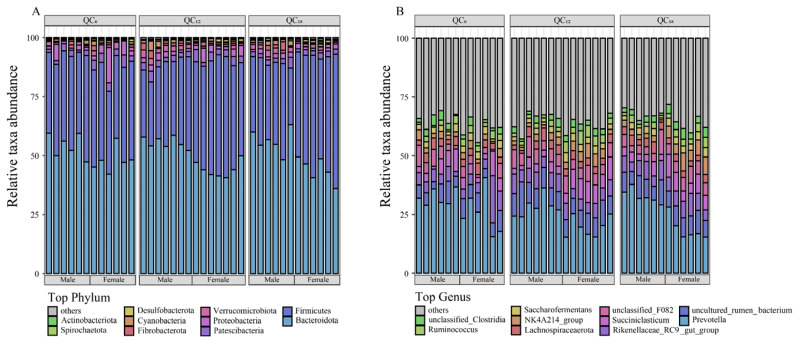
Rumen microbial composition. The stacked histogram showed the microbial composition at the phylum (A) and genus (B) levels.

In all samples, a total of 25 bacterial phyla and 486 bacterial genera were detected at the taxonomic level. Stacked histograms illustrate the microbial composition at the phylum and genus levels. Bacteroidota and Firmicutes emerged as the dominant phyla, with *Prevotella* as the dominant genus, displaying mean relative abundances of 50.3%, 39.7%, and 26.1%, respectively (Figures 2A, 2B).

**Figure 3:**
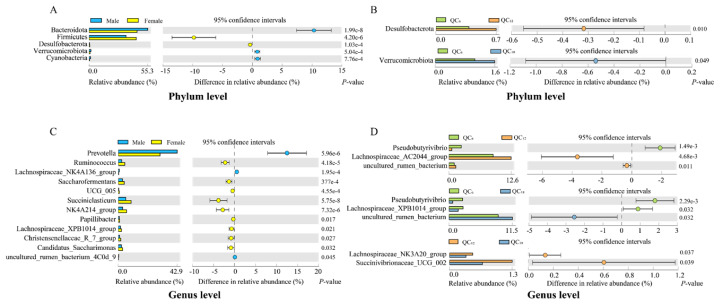
Analysis of gender and age differences within the rumen community. The extended bar chart illustrates the differences between genders (A, C) and ages (B, D) at the phylum and genus levels. The bar chart on the left showed the average relative abundance (%) of microbiota, while the dots on the right shows the average proportion of change (%) in the relative abundance of microorganisms within the 95% confidence interval, exclusively displaying microbiota with p<0.05.

Microbial differences at the phylum and genus levels were analyzed using STAMP. At the phylum level, the relative abundance of Bacteroidota, Verrucomicrobiota, and Cyanobacteria was significantly higher in the rumen of males than in females (p<0.05), whereas the relative abundance of Firmicutes and Desulfobacterota were significantly higher in females than in males (p<0.05, Figure 3A). Further, age-related analysis revealed that the relative abundance of Desulfobacterota in the rumen of QC_12_ was significantly higher than in QC_6_ (p = 0.01), and the relative abundance of Verrucomicrobiota in the rumen of QC_18_ was significantly higher than in QC_6_ (p = 0.049). There were no significant differences in the microbial composition at the phylum level between QC_12_ and QC_18_ (p>0.05, Figure 3B). At the genus level, the relative abundance of *Prevotella* and *Lachnospiraceae_NK4A136_group* was significantly higher in the rumen of males than in females (p<0.05). Conversely, the relative abundance of *Ruminococcus*, *Saccharofermentans*, *Succiniclasticum*, *Christensenellaceae_R_7_group*, and *Candidatus_Saccharimonas* was more abundant in females than in males (p<0.05, Figure 3C). Age difference analysis indicated a significant increase in the relative abundance of *Pseudobutyrivibrio* in the rumen of QC_6_, compared to QC_12_ and QC_18_ (p<0.01). Furthermore, the relative abundance of *Lachnospiraceae_NK3A20_group* and *Succinivibrionaceae_UCG_002* in the rumen of QC_12_ was significantly greater than in QC_18_ (p<0.05, Figure 3D).

Linear discriminant analysis (LDA, ≥4; p<0.05) effect size (LEfSe) and cladogram generated from the LEfSe analysis were performed to identify differentially abundant bacterial taxa in each group. Specifically, 9 bacterial genera were identified in females, 7 bacterial genera in males and 2 bacterial genera in QC_6_ (Supplements 2A, 2B). The Cladogram illustrates the most significant taxonomic differences among the groups (Supplement 2C, 2D). The Firmicute phylum, Prevotellaceae family, and Lachnospiraceae family were the most differentially abundant bacterial genera in females, males and QC_6_, respectively. There were no significant differences in microbial markers between the QC_12_ and QC_18_ groups.

### Microbial function prediction

**Figure 4:**
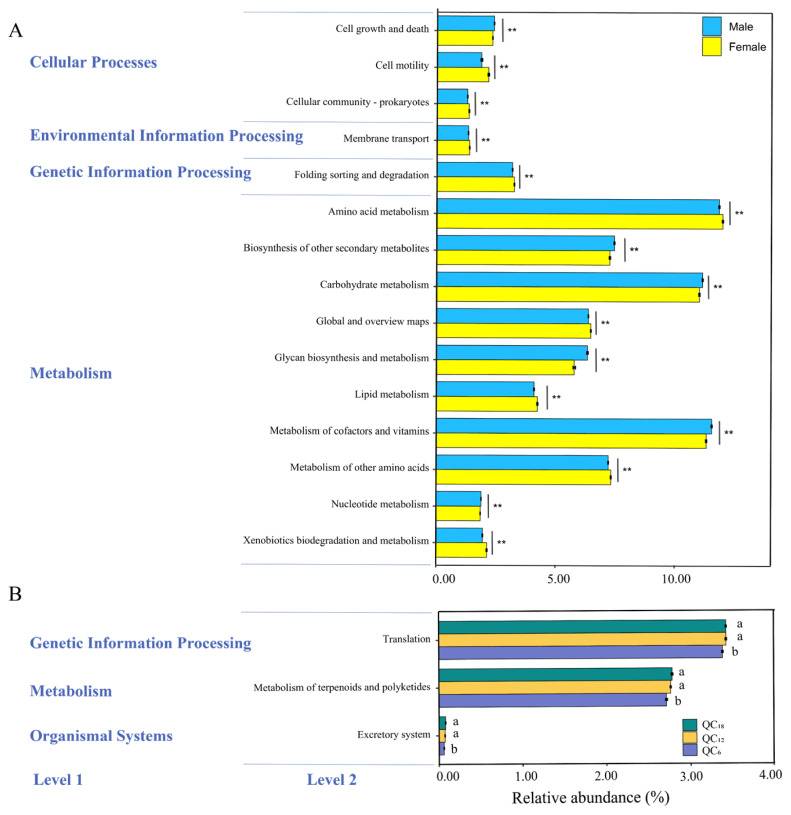
The 15 most significant KEGG pathways of rumen bacteria between males and females (A), and the 3 most significant KEGG pathways among age group of QC_6_, QC_12_, and QC_18_ (B). ** Indicates an extremely significant difference between males and females (p<0.01). ^a,b^ Different lowercase letters denoted significant differences among QC_6_, QC_12_, QC_18_ (p<0.05), while identical lowercase letters denote no significant differences (p>0.05). The standard error (SE) of mean was displayed with error bars.

To investigate how the functions of rumen bacteria vary with gender and age, we utilized PICRUSt2 to predict the functions of rumen bacteria. We then conducted an ANOVA followed by a T-test (males vs. females) and Duncan’s test (among QC_6_, QC_12_ and QC_18_) to compare the abundance of predicted KEGG pathways based on ASVs. A total of 45 KEGG pathways at level 2 were annotated, involving processes such as “cellular processes,” “genetic information processing,” “environmental information processing,” and “metabolism”. A comparison analysis of the KEGG pathways revealed the 15 most significant gender-related pathways with p-values below 0.01. Among them, five pathways related to the metabolism (biosynthesis of other secondary metabolites, carbohydrate metabolism, glycan biosynthesis and metabolism, metabolism of cofactors and vitamins, nucleotide metabolism) and one pathway related to cellular processes (cell growth and death) were more abundant in males than in females (p<0.05). On the contrary, five pathways related to metabolism (amino acid metabolism, global and overview maps, lipid metabolism, metabolism of other amino acids, xenobiotics biodegradation and metabolism), two pathways related to cellular processes (cell motility, cellular community-prokaryotes), one pathway related to environmental information processing (membrane transport) and one pathway related to genetic information processing (folding sorting and degradation) were more abundant in females than in males (p<0.05). In addition, three age-related differential pathways were identified (Figure 4B). Specifically, the relative abundance of predicted functions such as “translation,” “metabolism of terpenoids and polyketides,” and “excretory system” was significantly lower in the rumen of QC_6_, compared to QC_12_ and QC_18_ (p<0.05).

### Correlation analysis

**Figure 5:**
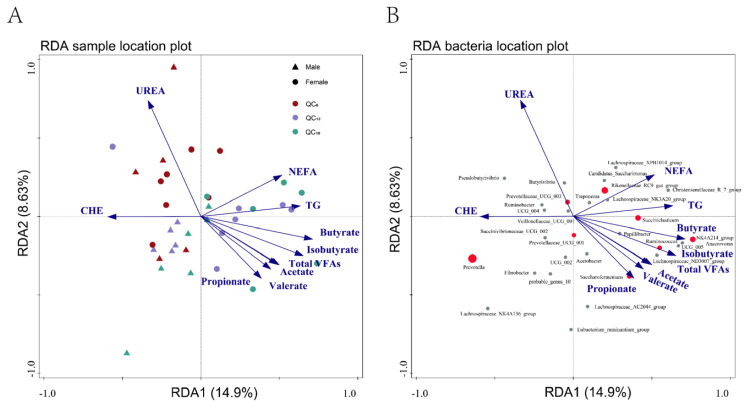
Redundancy analysis (RDA) illustrating the correlations between bacterial communities and physiological indices of rumen fermentation parameters and serum metabolites in Qinchuan cattle. (A) Each point represented a sample, while each arrow represents a quantitative explanatory variable (rumen fermentation parameters, serum metabites). The distance between two samples points approximates their differences in bacterial communities, and the cosine of the angles between explanatory variable arrows reflected their correlations. (B) Each point represented a bacterial genus; top 30 genera were represented. The size of each point reflected the abundance of the corresponding genus, with the top 8 genera highlight in red. Arrow length indicated the correlation between explanatory variables and the distribution of rumen bacterial communities at the genus level. Longer arrows signify stronger correlations. TG, Triglyceride; CHE, choline esterase; NEFA, nonestesterified fatty acid.

RDA was conducted to analyze the relationships between rumen bacteria, rumen fermentation parameters, and serum metabolites in Qinchuan cattle. The simple effects and conditional effects of explanatory variables on the rumen bacteria community structure after performing 2,000 permutations are presented in Supplement 3. Coloring the samples by gender and age revealed that the centroids of males and females were well separated, confirming a significant correlation between gender and the rumen bacteria community structure. However, the centroids of QC_12_ and QC_18_ were not separated, indicating a similarity in the rumen bacterial community (Figure 5A). RDA1 and RDA2 contributed 14.9% and 8.63%, respectively, to the variation in rumen bacterial community structure. Butyrate was the most significant factor associated with the bacterial community in the rumen, explaining 8.6% of the variation, followed by propionate (6.6%) and UA (5.7%)(conditional effects, p<0.05; Figure 5B; Supplement 3).

**Figure 6:**
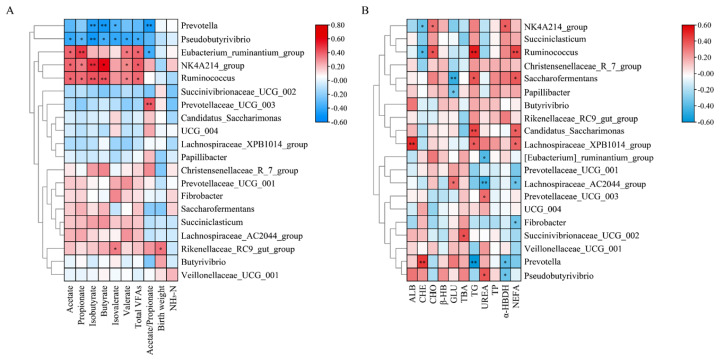
Relationships between main bacterial communities at the genus level and rumen fermentation parameters (A), and serum metabolites (B). Pearson rank correlations were represented by the color of each block in the heatmap, red indicated a positive correlation, while blue indicated a negative correlation. Top 20 bacteria genera were represented. ** and * indicate significance levels at 0.01 and 0.05, respectively. ALB, albumin; CHE, choline esterase; CHO, total cholesterol; β-Hb, β-hydroxybutyrate; GLU, glucose; TBA, total bile acid; TG, Triglyceride; TP, total protein; α-HBDH, α-hydroxybutyrate-dehydrogenase; NEFA, nonestesterified fatty acid.

The concentrations of acetate, propionate, valerate and total VFAs were positively correlated with *Ruminococcus* (*r* = 0.391, *r* = 0.372, *r* = 0.323, *r* = 0.380, respevtively), *NK4A214_group* (*r* = 0.389, *r* = 0.343, *r* = 0.342, *r* = 0.407), *Eubacterium_ruminantium_group* (*r* = 0.389, *r* = 0.481, *r* = 0.350, *r* = 0.388) and negatively correlated with *Pseudobutyrivibrio* (*r* = −22120.401, *r* = −0.322, *r* = −0.397, *r* = −0.375) (p<0.05). The concentrations of isobutyrate and butyrate were positively correlated with *Ruminococcus* (*r* = 0.423, *r* = 0.424), *NK4A214_group* (*r* = 0.517, *r* = 0.602), and negatively correlated with *Pseudobutyrivibrio* (*r* = −0.484, *r* = −0.367), *Prevotella* (*r* = −0.449, *r* = −0.476) (p<0.05). Furthermore, birth weight was positively correlated with *Rikenellaceae_RC9_gut_group* (*r* = 0.355) (Figure 6A; Supplement 4).

In serum, the concentration of ALB was positively correlated with *Lachnospiraceae_XPB1014_group* (*r* = 0.486); CHE was positively correlated with *Prevotella* (*r* = 0.461) and negatively correlated with *Ruminococcus* (*r* = −0.405), *NK4A214_group* (*r* = −0.357). GLU was negatively correlated with *Saccharofermentans* (*r* = −0.466); TG was positively correlated with *Ruminococcus* (*r* = 0.532), *Candidatus_Saccharimonas* (*r* = 0.426) and negatively correlated with *Prevotella* (*r* = −0.581). Nonesterifiedfatty acid (NEFA) positively correlated with *Ruminococcus* (*r* = 0.429) and negatively correlated with *Fibrobacter* (*r* = −0.338) (Figure 6B; Supplement 5).

## DISCUSSION

The production of ammonia nitrogen through the microbial deamination of feed proteins is essential for the digestion and utilization of nutrients in the rumen [20]. In this study, rumen ammonia nitrogen concentrations in Qinchuan cattle showed no significant differences across genders or ages. However, studies on Holstein cattle indicated a significantly higher rumen ammonia nitrogen concentration in males compared to females [21]. This is potentially due to differences in breed and diet composition.

VFAs are the primary energy source for ruminants, produced through the sequential degradation of feed nutrients by ruminal microbes and providing over 70% of their energy needs [1]. Acetate, propionate, and butyrate account for approximately 95% of the total VFAs. The proportions of acetate and propionate were significantly influenced by dietary composition, while the proportions of butyrate and valerate were less affected by diet [22]. Increasing the proportion of non-structural carbohydrates (NFC) in the diet typically decrease the acetate ratio and increase the propionate ratio [23]. In this study, all experimental cattle were fed the same diet, and no significant gender differences were observed in the concentrations of acetate and propionate. However, the proportion of acetate showed a trend toward increasing with age, potentially due to variations in dry matter intake (DMI). The acetate/propionate ratio did not show significant differences across genders or ages in Qinchuan cattle, suggesting similar ruminal fermentation patterns across these groups. Moreover, butyrate concentrations were significantly higher in females than in males, indicating clear gender differences. The concentrations of isobutyrate, butyrate, and isovalerate also increased with age (Table 1). Butyrate is produced by ruminal microbes through the fermentation of carbohydrates in the feed, such as insoluble cellulose and resistant starch, and is absorbed by the epithelial cells within the rumen. Most of the butyrate was subsequently converted into ketone bodies (β-HB), which serve as substrates for the synthesis of body fat [24]. An increase in butyrate concentration stimulated the expansion of the surface of rumen epithelial cells, and promoted the oxidation of short-chain fatty acids in the ketogenesis pathway [25]. Variations in butyrate concentration by gender and age may be attributed to differences in fat deposition capacities between males and females and to varying rates of fat deposition among different age groups of Qinchuan cattle. Isobutyrate and isovalerate, branched-chain VFAs produced from the fermentation of branched-chain amino acids, are crucial for the degradation of structural carbohydrates and for microbial protein synthesis in the rumen [26]. Supplementing branched-chain VFAs has been shown to enhance the production of ruminal microbes and increase cellulase activity in sheep [27]. In this study, the increase in branched-chain fatty acid concentrations in QC_18_ was likely related to the rise in total VFA concentrations.

In general, greater microbial community diversity correlates with enhanced stability of the community structure [28]. Current research suggested that sex hormones were a primary factor causing differences in gut microbiota between genders [29]. Research involving mice has shown that male mice had lower gut microbial diversity compared to female mice, influenced by hormonal variations. Castration eliminated these gender differences, while hormone replacement reinstated them, suggesting a direct hormonal effect on microbial composition [30]. However, research on older humans, such as postmenopausal women and elderly men, showed no significant gender differences in gut microbial diversity [31], suggesting that the observed gender differences in younger populations were influenced by fluctuating sex hormone levels. In our study, we observed no significant differences in the rumen microbial diversity between male and female Qinchuan cattle. This might be due to rumen microbial ecosystem in cattle might have responded differently to hormonal changes compared to the human or murine gut. The unique physiology of ruminants could lead to distinct interactions between hormones and microbiota. However, since the levels of sex hormones were not measured in this study, further research is needed to provide supporting evidence to elucidate the impact of sex hormones on rumen microbial diversity. Furthermore, rumen microbial diversity varied across different developmental stages. Studies on dairy cows have shown that both rumen microbial diversity and community similarity increased with age [10], which was consistent with our findings, indicating that age was one of the most important factors in the stability of rumen microbial community structure.

Firmicutes and Bacteroidetes were the two dominant phyla with the highest abundance in the rumen [32]. In this study, significant differences were observed in the abundance of these dominant phyla between males and females. The abundance of Bacteroidetes was significantly lower, whereas Firmicutes was significantly higher in females than in males (Figure 3). The ratio of Firmicutes to Bacteroidetes is crucial for maintaining normal gut homeostasis, and an increased ratio has been closely associated with the development of human obesity [33]. In addition, increased abundance of Firmicutes have been shown to be associated with higher feed efficiency and greater body fat accumulation in cattle [34,35]. This suggested that Firmicutes play a significant role in the regulation of body fat and the development of obesity. Generally, females have a higher percentage of body fat than males [12], which may be related to the increase in Firmicutes in females. Furthermore, Desulfobacteria is one of the main sulfate-reducing bacteria in the rumen, and the accumulation of its metabolites in the rumen epithelium may induce intestinal inflammation [36]. Akkermansia muciniphila, a member of Verrucomicrobia found in mammalian intestines, colonizes the intestinal mucosa and exhibits anti-inflammatory properties that protect the host from intestinal pathogens [37]. In our study, the decrease in Desulfobacteria in males may be related to the increase in Verrucomicrobia. Notably, there were also significant differences in Desulfobacteria and Verrucomicrobia among different age groups. Compared to QC_6_, Desulfobacteria significantly increased in QC_12_, while Verrucomicrobia exhibited a significant increase in QC_18_. These findings indicate dynamic changes in gut microbiota across different developmental stages of the host and highlight the beneficial role of Verrucomicrobia in promoting intestinal health.

Significant gender differences were observed in the abundance of *Prevotella*, *Ruminococcus*, *Saccharofermentans*, *Succiniclasticum* and *NK4A214_group*. *Prevotella*, which is widely present in the mammalian gastrointestinal tract, plays a crucial role in degrading carbohydrates through the fermentation of various plant polysaccharides [38]. *Prevotella* primarily produces propionate as a fermentation end-product. Once absorbed through the rumen wall, propionate serves as an important precursor for hepatic gluconeogenesis, playing a critical role in maintaining GLU homeostasis [39]. However, in our study, the propionate concentration in the rumen of male Qinchuan cattle did not show a significant change with the increase in *Prevotella* abundance. This might be related to the higher GLU utilization efficiency in males, meaning that the propionate produced by *Prevotella* was quickly converted to GLU and were utilized [40]. The significant enrichment of microbial functions related to glycan biosynthesis and metabolism in males also supported the finding. Butyrate was closely related to feed efficiency in ruminants, and researchers have observed a significant increase in butyrate concentration in Holstein Friesian dairy cows with high feed efficiency [41]. The higher TG levels were consistent with the higher body fat percentage and fat storage capacity typically observed in females [42]. Furthermore, females oxidize a greater proportion of lipids (EFA) for energy compared to carbohydrates and proteins than males. Additionally, females store more circulating free fatty acid (FFA) into adipose tissue during rest periods than males [43]. This aligns with our findings that the rumen microbiota in male Qinchuan cattle were significantly enriched in carbohydrate metabolism, while females were enriched in lipid metabolism. Moreover, *Ruminococcus* exhibited a significant positive correlation with the concentration of butyrate, and the levels of serum TG and NEFA (Figure 6). The higher levels of butyrate, TG, NEFA and increased abundance of *Ruminococcus* in females indicated that gender differences in feed efficiency and fat metabolism may be partly attributed to variations in the rumen microbiome composition, where *Ruminococcus* play key roles. *Ruminococcus*, *UCG-005*, *NK4A214_group*, *Saccharofermentans*, members of the Ruminococcaceae family, were famous for their robust fiber decomposition capabilities and were also primary butyrate producers [44]. The significant increase in these microbes in females suggested enhanced fiber degradation capabilities, enabling increased fermentation of plant fibers, which aligned with findings in Tibetan sheep [45]. In addition, we identified age-related differences in microbial abundance. In QC_6_, there was a notable increase in the abundance of *Pseudobutyrivibrio*, which is capable of fermenting various carbohydrates and producing butyrate as a key end product [46]. However, the concentration of butyrate in QC_6_ did not significantly improve. This aligns with previous findings, showing that variations in *Pseudobutyrivibrio* abundance did not correspond with changes in butyrate concentration [47]. The rumen microbial community exhibits significant functional redundancy, with multiple microorganisms capable of degrading the same substrates [48]. Alterations in community composition often do not lead to substantial changes in VFA concentrations [48,49]. The fluctuations in abundance of *Pseudobutyrivibrio* may not be sufficient to influence butyrate levels in the rumen. Notably, we did not observe any significant changes in the abundance of other butyrate-producing bacteria, suggesting that further investigation is necessary to fully understand these findings.

## CONCLUSION

This study explores the gender and age-related variations in serum metabolites, rumen fermentation performance, and microbiota composition in Qinchuan cattle. The concentration of butyrate in the females and QC_18_ was significantly higher than males and QC_6_, respectively. The content of GLU, TG, and NEFA in serum showed significant gender differences in Qinchuan cattle, with females exhibiting a decrease in GLU and increase in TG and EFA compared to males. Alpha diversity in the rumen bacterial community showed age-related variations, with no significant differences observed between genders. The relative abundance of *Prevotella* and *Lachnospiraceae_NK4A136_group* in rumen was lower in females, while *Ruminococcus*, *Succiniclasticum*, *NK4A214_group* and *Saccharofermentans* were significantly higher in females, compared to males. Functional prediction indicated that “amino acid metabolism” and “lipid metabolism” were mainly enriched in females, while “carbohydrate metabolism” and “glycan biosynthesis and metabolism” were enriched in males. RDA analysis suggested that butyrate was the most important factor related to the bacterial community in the rumen, with significant correlations observed with specific microbial genera. These results emphasize the complex relationships between in the rumen microbiota and the host, highlighting the importance of considering gender and age when evaluating ruminal health and function in cattle.

## Figures and Tables

**Table 1:** Composition and nutrient levels of diet for cattle (dry matter basis)

Items	Content
Ingredients, (%)	100
Corn silage	61.5
Wheat straw	10.0
Corn	15.1
Soybean meal	7.13
Wheat bran	4.28
NaCl	0.29
CaHPO_4_	0.29
Premix^1)^	5.00
Nutrient levels^2)^	
ME (MJ/kg)	10.4
OM (%)	95.8
CP (%)	9.53
NDF (%)	38.2
ADF (%)	21.9
EE (%)	3.15

1) The premix provided the following nutrients per kg of diets: VA, 8000 IU; VB_1_, 4 mg; VB_2_, 3.6 mg; VB_5_, 40 mg; VB_6_, 4 mg; VB_12_, 0.02 mg; VD_3_, 3000 IU; VE, 20 IU; VK_3_, 2 mg; biotin, 0.15 mg; folic acid, 1.0 mg; *D*-pantothenic acid, 11 mg; nicotinic acid, 10 mg; Cu (as copper sulfate), 10 mg; Fe (as ferrous sulfate), 80 mg; Mn (as manganese sulfate), 80 mg; Zn (as zinc sulfate), 75 mg; I (as potassium iodide), 0.40 mg; Se (as sodium selenite), 0.30 mg.

2) Metabolic energy (ME) was a calculated value, whereas the others were measured values.

ME, metabolizable energy; OM, organic matter; CP, crude protein; NDF, neutral detergent fiber; ADF, acid detergent fiber; EE, Ether extract.

**Table 2:** Analysis of gender and age differences in rumen fermentation performance

Item	Gender		Age			p-value		
		
	Males	Females	QC_6_	QC_12_	QC_18_	Gender	Age	Gender×Age
Birth weight (kg)	21.1±0.57	20.5±0.70	20.3±0.79	20.6±0.47	21.6±1.02	0.449	0.495	0.330
NH_3_-N (mg/L)	4.47±0.36	4.42±0.33	4.23±0.27	4.20±0.39	4.95±0.54	0.929	0.429	0.964
Concentration of VFAs (mmol/L)								
Acetate	79.9±7.36	94.6±7.09	74.3±10.3	85.0±7.03	103±8.21	0.134	0.081	0.225
Propionate	21.8±1.89	25.6±1.95	21.1±2.86	22.5±1.97	27.7±1.98	0.148	0.124	0.151
Isobutyrate	0.85±0.06	1.01±0.08	0.75±0.09^b^	0.95±0.08^ab^	1.09±0.08^a^	0.127	0.030	0.942
But yrate	9.67±0.81^B^	13.1±1.21^A^	9.33±1.36^b^	10.8±1.15^b^	14.1±1.88^a^	0.023	0.036	0.916
Isovalerate	1.61±0.15	1.73±0.15	1.31±0.22^b^	1.73±0.14^ab^	1.96±0.15^a^	0.485	0.049	0.194
Valerate	1.41±0.14	1.54±0.14	1.25±0.19	1.44±0.13	1.73±0.16	0.441	0.141	0.296
Total VFAs	117±9.69	138±10.4	111±14.0	122±10.3	150±11.4	0.136	0.089	0.315
Acetate/Propionate	3.67±0.07	3.72±0.08	3.54±0.08	3.80±0.08	3.71±0.11	0.615	0.143	0.429

A,B Within the same row, distinct capital letter superscripts indicate significant differences between males and females (p<0.05).

a,b Differing lowercase letter superscripts denote significant differences among QC_6_, QC_12_, QC_18_ (p<0.05), while identical lowercase superscripts indicate no significant differences (p>0.05).

NH_3_-N, ammonia nitrogen; VFAs, volatile fatty acids.

**Table 3:** Analysis of gender and age differences in serum metabolic indicates

Item	Gender		Age			p-value		
		
	Males	Females	QC_6_	QC_12_	QC_18_	Gender	Age	Gender×Age
ALB (g/L)	25.2±3.18	25.7±2.93	28.2±1.89^a^	24.5±2.88^b^	23.7±2.13^b^	0.579	<0.001	0.710
CHE (U/L)	442±68.3^A^	387±69.1^B^	422±72.1	400±50.5	424±97.0	0.023	0.624	0.414
CHO (mmol/L)	2.61±0.7^B^	3.27±0.61^A^	2.70±0.83	3.04±0.82	3.06±0.52	0.006	0.369	0.893
β-HB (mmol/L)	0.37±0.07	0.36±0.08	0.36±0.08	0.35±0.07	0.39±0.06	0.500	0.440	0.199
GLU (mmol/L)	5.06±1.28^A^	3.98±1.42^B^	4.41±1.01	4.83±1.94	4.26±1.16	0.026	0.547	0.475
TBA (umol/L)	18.0±4.78	15.9±8.18	20.1±7.24	17.1±6.41	13.8±5.28	0.373	0.059	0.168
TG (mmol/L)	0.23±0.04^B^	0.37±0.08^A^	0.29±0.09	0.31±0.12	0.30±0.08	<0.001	0.859	0.327
UA (mmol/L)	2.92±0.84	2.72±0.72	3.45±0.87^a^	2.64±0.54^b^	2.39±0.53^b^	0.373	0.001	0.997
TP (g/L)	60.4±9.53	61.2±5.61	63.0±4.60	57.2±6.44	62.7±10.2	0.752	0.113	0.990
α-HBDH (U/L)	919±178	1033±209	903±135	945±120	1085±281	0.062	0.055	0.715
NEFA m(mol/L)	0.05±0.05^B^	0.15±0.14^A^	0.08±0.06	0.12±0.18	0.08±0.06	0.009	0.571	0.168

A,B Within the same row, distinct capital letter superscripts indicate significant differences between males and females (p<0.05).

a,b Differing lowercase letter superscripts denote significant differences among QC_6_, QC_12_, QC_18_ (p<0.05), while identical lowercase superscripts indicate no significant differences (p>0.05).

ALB, albumin; CHE, cholinesterase; CHO, total cholesterol; β-HB, β-hydroxybutyrate; GLU, glucose; TBA, total bile acid; TG, Triglyceride; UA, urea; TP, total protein; α-HBDH, α-hydroxybutyrate-dehydrogenase; NEFA, nonestesterified fatty acid.

## SUPPLEMENTARY MATERIAL

**Supplement 1**. The rarefaction curves for all samples.

**Supplement 2**. Comparison of bacterial communities and function prediction in different groups.

**Supplement 3**. Effect of explanatory variables on the species composition in rumen of Qinchuan cattle

**Supplement 4**. Pearson correlation coefficients (r) between rumen fermentation index and dominant bacteria in rumen

**Supplement 5**. Pearson correlation coefficients (r) between serum metabolites and dominant bacteria in rumen

## References

[1] Russell JB, Rychlik JL (2001). Factors that alter rumen microbial ecology. Science.

[2] Zilber-Rosenberg I, Rosenberg E (2008). Role of microorganisms in the evolution of animals and plants: the hologenome theory of evolution. FEMS Microbiol Rev.

[3] Lima J, Auffret MD, Stewart RD (2019). Identification of rumen microbial genes involved in pathways linked to appetite, growth, and feed conversion efficiency in cattle. Front Genet.

[4] Yin X, Ji S, Duan C (2021). Age-related changes in the ruminal microbiota and their relationship with rumen fermentation in lambs. Front Microbiol.

[5] Bolnick DI, Snowberg LK, Hirsch PE (2014). Individual diet has sex-dependent effects on vertebrate gut microbiota. Nat Commun.

[6] Sinha T, Vich Vila A, Garmaeva S (2019). Analysis of 1135 gut metagenomes identifies sex-specific resistome profiles. Gut Microbes.

[7] Zhang X, Zhong H, Li Y (2021). Sex- and age-related trajectories of the adult human gut microbiota shared across populations of different ethnicities. Nat Aging.

[8] Markle JGM, Frank DN, Mortin-Toth S (2013). Sex differences in the gut microbiome drive hormone-dependent regulation of autoimmunity. Science.

[9] Beharka AA, Nagaraja TG, Morrill JL, Kennedy GA, Klemm RD (1998). Effects of form of the diet on anatomical, microbial, and fermentative development of the rumen of neonatal calves1. J Dairy Sci.

[10] Jami E, Israel A, Kotser A, Mizrahi I (2013). Exploring the bovine rumen bacterial community from birth to adulthood. ISME J.

[11] Zhang G, Wang Y, Luo H (2019). The association between inflammaging and age-related changes in the ruminal and fecal microbiota among lactating holstein cows. Front Microbiol.

[12] Pogorzelska-Przybyłek P, Nogalski Z, Sobczuk-Szul M, Momot M (2021). The effect of gender status on the growth performance, carcass and meat quality traits of young crossbred Holstein-Friesian×Limousin cattle. Anim Biosci.

[13] Hou M, Ye M, Ma X (2024). Colon microbiota and metabolite potential impact on tail fat deposition of Altay sheep. Microbiol Spectr.

[14] Holman DB, Gzyl KE, Scott H, Cara S, Prieto N, López-Campos Ó (2024). Associations between the rumen microbiota and carcass merit and meat quality in beef cattle. Appl Microbiol Biotechnol.

[15] Van Soest PJ, Robertson JB, Lewis BA (1991). Methods for dietary fiber, neutral detergent fiber, and nonstarch polysaccharides in relation to animal nutrition. J Dairy Sci.

[16] Callahan BJ, McMurdie PJ, Rosen MJ, Han AW, Johnson AJA, Holmes SP (2016). DADA2: high-resolution sample inference from Illumina amplicon data. Nat Methods.

[17] Bolyen E, Rideout JR, Dillon MR (2019). Reproducible, interactive, scalable and extensible microbiome data science using QIIME 2. Nat Biotechnol.

[18] Parks DH, Tyson GW, Hugenholtz P, Beiko RG (2014). STAMP: statistical analysis of taxonomic and functional profiles. Bioinformatics.

[19] Douglas GM, Maffei VJ, Zaneveld JR (2020). PICRUSt2 for prediction of metagenome functions. Nat Biotechnol.

[20] Ceconi I, Ruiz-Moreno MJ, DiLorenzo N, DiCostanzo A, Crawford GI (2015). Effect of urea inclusion in diets containing corn dried distillers grains on feedlot cattle performance, carcass characteristics, ruminal fermentation, total tract digestibility, and purine derivatives-to-creatinine index. J Anim Sci.

[21] He Y, Wang H, Yu Z (2018). Effects of the gender differences in cattle rumen fermentation on anaerobic fermentation of wheat straw. J Clean Prod.

[22] Zhu X, Liu B, Xiao J (2022). Effects of different roughage diets on fattening performance, meat quality, fatty acid composition, and rumen microbe in steers. Front Nutr.

[23] Penner GB, Oba M (2009). Increasing dietary sugar concentration may improve dry matter intake, ruminal fermentation, and productivity of dairy cows in the postpartum phase of the transition period. J Dairy Sci.

[24] Baaske L, Gäbel G, Dengler F (2020). Ruminal epithelium: a checkpoint for cattle health. J Dairy Res.

[25] Niwińska B, Hanczakowska E, Arciszewski MB, Klebaniuk R (2017). Review: Exogenous butyrate: implications for the functional development of ruminal epithelium and calf performance. Animal.

[26] Wang C, Liu Q, Zhang YL (2015). Effects of isobutyrate supplementation on ruminal microflora, rumen enzyme activities and methane emissions in Simmental steers. J Anim Physiol Anim Nutr (Berl).

[27] Moharrery A, Das TK (2001). Correlation between microbial enzyme activities in the rumen fluid of sheep under different treatments. Reprod Nutr Dev.

[28] Ptacnik R, Solimini AG, Andersen T (2008). Diversity predicts stability and resource use efficiency in natural phytoplankton communities. Proc Natl Acad Sci USA.

[29] Neuman H, Debelius JW, Knight R, Koren O (2015). Microbial endocrinology: the interplay between the microbiota and the endocrine system. FEMS Microbiol Rev.

[30] Yurkovetskiy L, Burrows M, Khan AA (2013). Gender bias in autoimmunity is influenced by microbiota. Immunity.

[31] Haro C, Rangel-Zúñiga OA, Alcalá-Díaz JF (2016). Intestinal Microbiota is influenced by gender and body mass index. PLOS ONE.

[32] Mizrahi I, Wallace RJ, Moraïs S (2021). The rumen microbiome: balancing food security and environmental impacts. Nat Rev Microbiol.

[33] Stojanov S, Berlec A, Štrukelj B (2020). The influence of probiotics on the firmicutes/bacteroidetes ratio in the treatment of obesity and inflammatory bowel disease. Microorganisms.

[34] Myer PR, Smith TPL, Wells JE, Kuehn LA, Freetly HC (2015). Rumen microbiome from steers differing in feed efficiency. PLOS ONE.

[35] Wang H, He Y, Li H (2019). Rumen fermentation, intramuscular fat fatty acid profiles and related rumen bacterial populations of Holstein bulls fed diets with different energy levels. Appl Microbiol Biotechnol.

[36] Kushkevych I, Dordević D, Vítězová M (2021). Possible synergy effect of hydrogen sulfide and acetate produced by sulfate-reducing bacteria on inflammatory bowel disease development. J Adv Res.

[37] Reid G, Younes JA, Van der Mei HC, Gloor GB, Knight R, Busscher HJ (2011). Microbiota restoration: natural and supplemented recovery of human microbial communities. Nat Rev Microbiol.

[38] Accetto T, Avguštin G (2019). The diverse and extensive plant polysaccharide degradative apparatuses of the rumen and hindgut Prevotella species: A factor in their ubiquity?. Syst Appl Microbiol.

[39] De Vadder F, Kovatcheva-Datchary P, Goncalves D (2014). Microbiota-generated metabolites promote metabolic benefits via gut-brain neural circuits. Cell.

[40] Trotta RJ, Harmon DL, Matthews JC, Swanson KC (2022). Nutritional and physiological constraints contributing to limitations in small intestinal starch digestion and glucose absorption in ruminants. Ruminants.

[41] Shabat SKB, Sasson G, Doron-Faigenboim A (2016). Specific microbiome-dependent mechanisms underlie the energy harvest efficiency of ruminants. ISME J.

[42] Blaak E (2001). Gender differences in fat metabolism. Curr Opin Clin Nutr Metab Care.

[43] Mauvais-Jarvis F (2024). Sex differences in energy metabolism: natural selection, mechanisms and consequences. Nat Rev Nephrol.

[44] Vital M, Howe AC, Tiedje JM (2014). Revealing the Bacterial butyrate synthesis pathways by analyzing (meta)genomic data. mBio.

[45] Guo X, Sha Y, Lv W (2022). Sex differences in rumen fermentation and microbiota of Tibetan goat. Microb Cell Fact.

[46] Van Gylswyk NO, Hippe H, Rainey FA (1996). Pseudobutyrivibrio ruminis gen. nov., sp. nov., a butyrate-producing bacterium from the rumen that closely resembles Butyrivibrio fibrisolvens in phenotype. Int J Syst Evol Microbiol.

[47] Xue Y, Lin L, Hu F, Zhu W, Mao S (2020). Disruption of ruminal homeostasis by malnutrition involved in systemic ruminal microbiota-host interactions in a pregnant sheep model. Microbiome.

[48] Weimer PJ (2015). Redundancy, resilience, and host specificity of the ruminal microbiota: implications for engineering improved ruminal fermentations. Front Microbiol.

[49] Welkie DG, Stevenson DM, Weimer PJ (2010). ARISA analysis of ruminal bacterial community dynamics in lactating dairy cows during the feeding cycle. Anaerobe.

