# Supplementary figures and images for: Gender and age-related variations in rumen fermentation and microbiota of Qinchuan cattle
Source: Anim Biosci. 2024 Oct 24;38(5):941–54. doi: 10.5713/ab.24.0328 (PMC12062828; doi:10.5713/ab.24.0328)

16 1. Supplement 1.

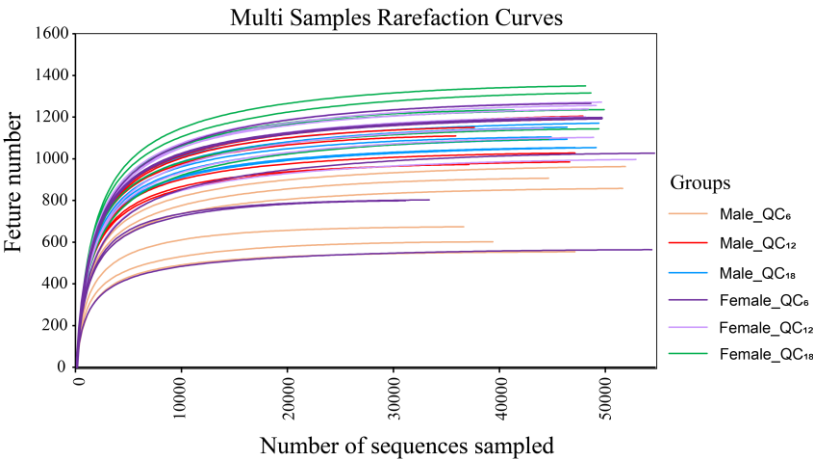

17

18

Supplement 1. The rarefaction curves for all samples.

19

Supplement: Supplementary file 1 [file ab-24-0328-Supplementary-1.pdf]
