# Supplementary material for: Gender and age-related variations in rumen fermentation and microbiota of Qinchuan cattle
Source: Anim Biosci. 2024 Oct 24;38(5):941–54. doi: 10.5713/ab.24.0328 (PMC12062828; doi:10.5713/ab.24.0328)
Supplement: Supplementary file 2 [file ab-24-0328-Supplementary-2.pdf]

2. Supplement 2.

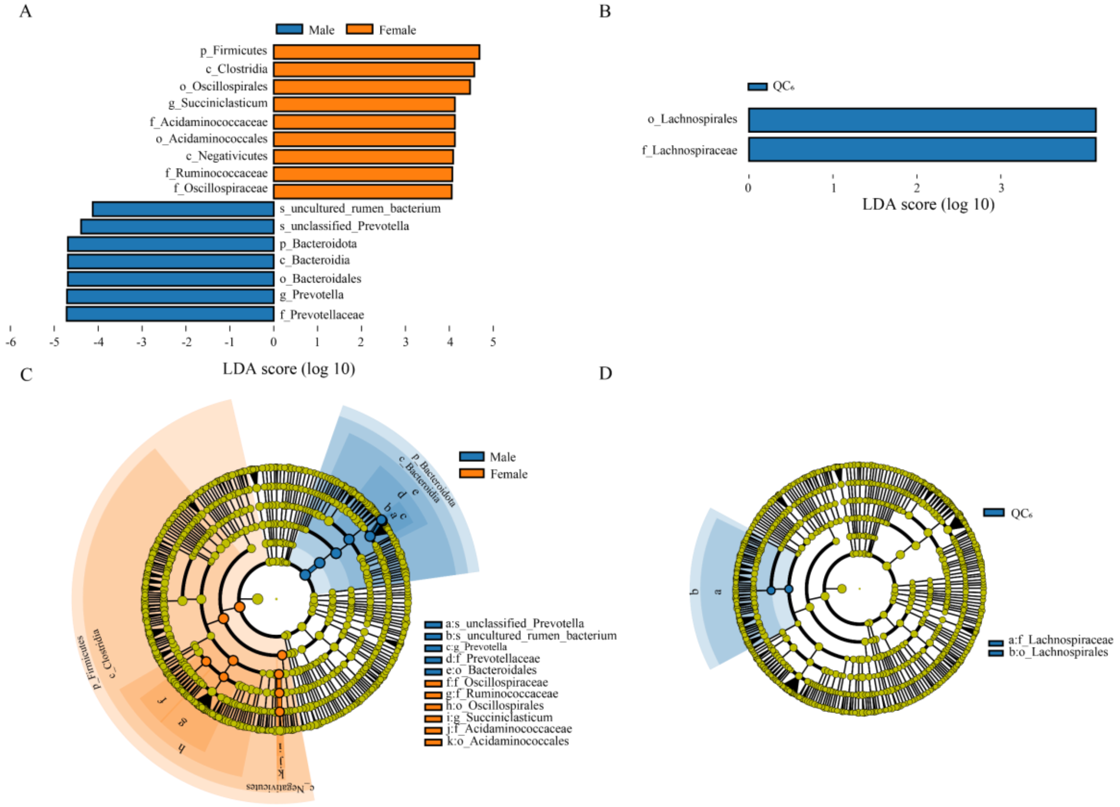

Supplement 2. Comparison of bacterial communities and function prediction in different groups. (A, C) Linear discriminant analysis (LDA, with a threshold of |log10 LDA|≥ 4; P < 0.05; Wilcoxon test) score derived from LEfSe (Linear discriminant analysis effect size) analysis identified the genera of which abundances significantly differed in each groups; the level represented in both sides as p, phylum; c, class; o, order; f, family; g, genus. (B, D) Cladogram generated from LEfSe analysis showed the relationship between taxon (the levels represent, from the inner to outer rings, phylum, class, order, family, and genus). Differences were represented by the color of the group where taxa were most abundant; Deep blue, taxa abundant in QCM (A) and QC6 (C); Red, taxa abundant in QCF (A).
