# Supplementary material for: Gender and age-related variations in rumen fermentation and microbiota of Qinchuan cattle
Source: Anim Biosci. 2024 Oct 24;38(5):941–54. doi: 10.5713/ab.24.0328 (PMC12062828; doi:10.5713/ab.24.0328)
Supplement: Supplementary file 3 [file ab-24-0328-Supplementary-3.pdf]

3. Supplement 3.

Supplement 3. Effect of explanatory variables on the species composition in rumen of Qinchuan cattle.

Name	Explains %	Pseudo-F	*P*-value
Simple effects:			
Butyrate	8.6	3.2	0.001
Isobutyrate	8.0	3.0	0.001
TG	7.7	2.8	0.002
UREA	7.2	2.6	0.004
CHE	6.2	2.2	0.014
NEFA	6.1	2.2	0.009
Total VFAs	5.9	2.1	0.015
Acetate	5.7	2.1	0.019
Valerate	5.5	2.0	0.026
Propionate	4.9	1.7	0.047
Conditional effects			
Butyrate	8.6	3.2	0.001
Isobutyrate	2.1	0.9	0.572
TG	3.2	1.3	0.184
UREA	5.7	2.3	0.010
CHE	1.7	0.7	0.757
NEFA	3.9	1.6	0.068
Total VFAs	2.5	1.1	0.373
Acetate	3.9	1.7	0.062
Valerate	1.9	0.8	0.630
Propionate	6.9	2.7	0.001

Note: Explanatory variables with simple effects *P*-values < 0.05 were represented. TG, Triglyceride; CHE, choline esterase; NEFA, nonestesterified fatty acid.
