# Supplementary material for: Gender and age-related variations in rumen fermentation and microbiota of Qinchuan cattle
Source: Anim Biosci. 2024 Oct 24;38(5):941–54. doi: 10.5713/ab.24.0328 (PMC12062828; doi:10.5713/ab.24.0328)
Supplement: Supplementary file 4 [file ab-24-0328-Supplementary-4.pdf]

## 4. Supplement 4

Supplement 4. Pearson correlation coefficients (r) between rumen fermentation index and dominant bacteria in rumen.

| Id | | Acetate | Propionate | Isobutyrate | Butyrate | Isovalerate | Valerate | Total VFAs | Acetate/ Propionate | Birth weight |
|---|---|---|---|---|---|---|---|---|---|---|
| *Prevotella* | *r* | -0.312 | -0.210 | -0.449 | -0.476 | -0.355 | -0.263 | -0.303 | -0.470 | 0.027 |
| | *P*-value | 0.056 | 0.207 | 0.005 | 0.002 | 0.029 | 0.111 | 0.065 | 0.003 | 0.872 |
| *Rikenellaceae_RC9_gut_group* | *r* | 0.191 | 0.132 | 0.176 | 0.233 | 0.339 | 0.137 | 0.190 | 0.280 | 0.355 |
| | *P*-value | 0.251 | 0.431 | 0.291 | 0.159 | 0.037 | 0.411 | 0.253 | 0.089 | 0.029 |
| *Succiniclasticum* | *r* | 0.180 | 0.201 | 0.261 | 0.290 | 0.174 | 0.203 | 0.178 | -0.094 | -0.063 |
| | *P*-value | 0.280 | 0.225 | 0.113 | 0.077 | 0.297 | 0.222 | 0.286 | 0.576 | 0.708 |
| *NK4A214_group* | *r* | 0.389 | 0.343 | 0.517 | 0.602 | 0.255 | 0.342 | 0.407 | 0.232 | -0.151 |
| | *P*-value | 0.016 | 0.035 | 0.001 | <0.001 | 0.123 | 0.036 | 0.011 | 0.161 | 0.366 |
| *Saccharofermentans* | *r* | 0.165 | 0.221 | 0.126 | 0.212 | -0.075 | 0.060 | 0.183 | -0.208 | -0.222 |
| | *P*-value | 0.321 | 0.182 | 0.450 | 0.201 | 0.653 | 0.722 | 0.272 | 0.210 | 0.181 |
| *Ruminococcus* | *r* | 0.391 | 0.372 | 0.423 | 0.424 | 0.284 | 0.323 | 0.380 | 0.065 | -0.029 |
| | *P*-value | 0.015 | 0.021 | 0.008 | 0.008 | 0.084 | 0.048 | 0.019 | 0.699 | 0.863 |
| *Prevotellaceae_UCG_001* | *r* | 0.173 | 0.166 | 0.080 | 0.056 | 0.236 | 0.292 | 0.166 | 0.093 | -0.088 |
| | *P*-value | 0.300 | 0.319 | 0.632 | 0.735 | 0.155 | 0.075 | 0.319 | 0.579 | 0.598 |
| *Prevotellaceae_UCG_003* | *r* | -0.117 | -0.204 | -0.110 | -0.058 | -0.109 | -0.203 | -0.150 | 0.415 | 0.109 |
| | *P*-value | 0.486 | 0.219 | 0.511 | 0.729 | 0.514 | 0.221 | 0.369 | 0.010 | 0.516 |
| *Candidatus_Saccharimonas* | *r* | -0.031 | -0.070 | -0.034 | -0.009 | 0.148 | -0.070 | -0.018 | 0.179 | 0.058 |
| | *P*-value | 0.854 | 0.677 | 0.839 | 0.955 | 0.376 | 0.677 | 0.913 | 0.282 | 0.728 |
| *Lachnospiraceae_XPB1014_group* | *r* | -0.122 | -0.127 | -0.097 | 0.030 | -161 | -0.186 | -0.081 | 0.035 | 0.038 |
| | *P*-value | 0.464 | 0.447 | 0.563 | 0.857 | 0.333 | 0.264 | 0.627 | 0.835 | 0.820 |
| *Butyrivibrio* | *r* | 0.012 | 0.070 | -0.037 | 0.040 | 0.097 | -0.017 | 0.036 | -0.227 | 0.257 |
| | *P*-value | 0.942 | 0.677 | 0.827 | 0.811 | 0.561 | 0.918 | 0.830 | 0.171 | 0.119 |
| *Christensenellaceae_R_7_group* | *r* | 0.106 | 0.036 | 0.266 | 0.316 | 0.017 | 0.097 | 0.146 | 0.305 | -0.225 |
| | *P*-value | 0.526 | 0.829 | 0.107 | 0.053 | 0.917 | 0.564 | 0.383 | 0.063 | 0.173 |
| *Fibrobacter* | *r* | 0.139 | 0.152 | 0.055 | 0.028 | 0.314 | 0.155 | 0.119 | 0.017 | -0.005 |
| | *P*-value | 0.405 | 0.362 | 0.744 | 0.865 | 0.055 | 0.354 | 0.475 | 0.918 | 0.975 |
| *Succinivibrionaceae_UCG_002* | *r* | -0.130 | -0.094 | --0.162 | -0.224 | -0.181 | -0.107 | -0.150 | -0.233 | 0.127 |
| | *P*-value | 0.436 | 0.573 | 0.332 | 0.176 | 0.276 | 0.524 | 0.368 | 0.160 | 0.447 |
| *Eubacterium_ruminantium_group* | *r* | 0.389 | 0.481 | 0.216 | 0.208 | 0.124 | 0.350 | 0.388 | -0.374 | -0.030 |
| | *P*-value | 0.016 | 0.002 | 0.194 | 0.211 | 0.457 | 0.031 | 0.016 | 0.021 | 0.860 |
| *Lachnospiraceae_AC2044_group* | *r* | 0.218 | 0.243 | 0.145 | 0.105 | 0.085 | 0.290 | 0.199 | -0.084 | -0.040 |
| | *P*-value | 0.189 | 0.142 | 0.384 | 0.532 | 0.613 | 0.077 | 0.231 | 0.617 | 0.811 |
| *Pseudobutyrivibrio* | *r* | -0.401 | -0.322 | -0.484 | -0.367 | -0.403 | -0.397 | -0.375 | -0.316 | -0.099 |
| | *P*-value | 0.013 | 0.049 | 0.002 | 0.023 | 0.012 | 0.014 | 0.020 | 0.053 | 0.555 |
| *Papillibacter* | *r* | -0.012 | -0.061 | -0.056 | -0.022 | -0.124 | -0.037 | 0.033 | 0.201 | -0.189 |

| | | | | | | | | | | |
|---|---|---|---|---|---|---|---|---|---|---|
| | *P*-value | 0.943 | 0.716 | 0.737 | 0.897 | 0.458 | 0.828 | 0.844 | 0.227 | 0.256 |
| *UCG_004* | *r* | -0.040 | -0.095 | -0.039 | -0.111 | 0.138 | -0.088 | -0.066 | 0.240 | 0.026 |
| | *P*-value | 0.810 | 0.571 | 0.818 | 0.506 | 0.410 | 0.598 | 0.694 | 0.147 | 0.878 |
| *Veillonellaceae_UCG_001* | *r* | 0.007 | 0.010 | 0.025 | 0.084 | 0.034 | 0.053 | -0.010 | 0.043 | 0.125 |
| | *P*-value | 0.965 | 0.953 | 0.883 | 0.618 | 0.841 | 0.750 | 0.953 | 0.797 | 0.454 |
