# Supplementary material for: Gender and age-related variations in rumen fermentation and microbiota of Qinchuan cattle
Source: Anim Biosci. 2024 Oct 24;38(5):941–54. doi: 10.5713/ab.24.0328 (PMC12062828; doi:10.5713/ab.24.0328)
Supplement: Supplementary file 5 [file ab-24-0328-Supplementary-5.pdf]

5. Supplement 5.

Supplement 5. Pearson correlation coefficients (r) between serum metabolites and dominant bacteria in rumen.

Id		ALB	CHE	CHO	β-HB	GLU	TBA	TG	UREA	TP	α-HBDH	EFA
Prevotella	*r*	0.098	0.461	-0.207	0.074	0.301	0.177	-0.581	0.170	-0.058	-0.382	-0.312
	P-value	0.185	0.004	0.213	0.659	0.066	0.302	<0.001	0.728	0.728	0.018	0.057
Rikenellaceae_RC9_gut_group	*r*	0.029	-0.119	-0.108	0.023	0.008	-0.256	0.240	0.048	0.140	0.289	0.049
	P-value	0.865	0.477	0.517	0.891	0.960	0.132	0.146	0.773	0.402	0.079	0.769
Succiniclasticum	*r*	0.009	-0.293	0.127	-0.023	-0.053	-0.296	0.221	-0.219	0.002	0.238	0.175
	P-value	0.957	0.075	0.190	0.890	0.754	0.079	0.182	0.186	0.992	0.151	0.294
NK4A214_group	*r*	-0.229	-0.357	0.325	0.165	-0.299	-0.178	0.284	-0.171	0.035	0.367	0.130
	P-value	0.166	0.028	0.047	0.323	0.068	0.300	0.083	0.305	0.833	0.024	0.438
Saccharofermentans	*r*	0.014	-0.042	0.279	0.154	-0.466	-0.024	0.388	-0.074	0.262	0.306	0.380
	P-value	0.934	0.804	0.090	0.356	0.003	0.888	0.016	0.657	0.112	0.062	0.019
Ruminococcus	*r*	-0.088	-0.405	0.395	0.020	-0.148	-0.154	0.532	-0.248	-0.004	0.267	0.429
	P-value	0.598	0.012	0.014	0.905	0.375	0.371	0.001	0.134	0.982	0.104	0.007
Prevotellaceae_UCG_001	*r*	-0.111	0.024	0.119	0.021	0.205	0.148	-0.269	-0.123	-0.121	-0.052	-0.317
	P-value	0.507	0.887	0.476	0.899	0.217	0.389	0.103	0.462	0.468	0.755	0.053
Prevotellaceae_UCG_003	*r*	0.007	0.026	-0.181	-0.180	-0.124	0.120	-0.026	0.333	-0.077	-0.042	0.026
	P-value	0.968	0.875	0.278	0.280	0.460	0.486	0.877	0.041	0.644	0.804	0.877
Candidatus_Saccharimonas	*r*	0.153	-0.195	0.048	0.008	0.050	-0.304	0.426	0.033	-0.019	-0.051	0.364
	P-value	0.359	0.240	0.776	0.961	0.765	0.072	0.008	0.842	0.909	0.761	0.025
Lachnospiraceae_XPB1014_group	*r*	0.486	-0.188	0.079	0.029	-0.079	-0.107	0.372	0.289	0.225	0.088	0.346
	P-value	0.002	0.259	0.638	0.864	0.636	0.533	0.022	0.079	0.175	0.599	0.033
Butyrivibrio	*r*	0.305	-0.103	-0.130	0.132	0.113	-0.081	0.178	0.127	0.124	-0.029	0.017
	P-value	0.063	0.540	0.436	0.428	0.498	0.637	0.285	0.448	0.459	0.865	0.920
Christensenellaceae_R_7_group	*r*	0.060	-0.265	0.090	0.143	-0.224	-0.110	0.227	0.142	0.169	0.174	0.122
	P-value	0.721	0.107	0.592	0.393	0.177	0.522	0.171	0.395	0.310	0.295	0.464
Fibrobacter	*r*	-0.227	0.147	-0.234	-0.117	0.027	0.215	-0.175	-0.213	-0.205	-0.119	-0.338
	P-value	0.171	0.377	0.157	0.486	0.874	0.209	0.294	0.198	0.218	0.478	0.038
Succinivibrionaceae_UCG_002	*r*	0.156	0.131	-0.115	-0.182	0.115	0.399	-0.008	-0.033	0.017	-0.132	-0.227
	P-value	0.348	0.434	0.492	0.274	0.491	0.016	0.963	0.846	0.919	0429	0.171
Eubacterium_ruminantium_group	*r*	-0.169	0.071	0.301	0.114	-0.028	0.187	-0.018	-0.405	-0.007	0.070	-0.067
	P-value	0.309	0.670	0.066	0.494	0.867	0.278	0.919	0.012	0.969	0.675	0.688
Lachnospiraceae_AC2044_group	*r*	-0.051	-0.159	0.121	-0.180	0.328	0.072	-0.310	-0.450	-0.153	-0.076	-0.352
	P-value	0.762	0.339	0.471	0.281	0.045	0.676	0.058	0.005	0.359	0.650	0.030
Pseudobutyrivibrio	*r*	0.310	0.241	-0.206	0.243	0.045	0.272	-0.236	0.388	0.074	-0.349	-0.098
	P-value	0.059	0.145	0.216	0.141	0.789	0.109	0.153	0.016	0.657	0.032	0.559
Papillibacter	*r*	0.093	-0.034	0.108	0.148	-0.340	0.045	0.205	-0.112	0.217	0.147	0.187
	P-value	0.580	0.839	0.517	0.376	0.037	0.796	0.217	0.505	0.190	0.377	0.260

UCG_004	*r*	-0.128	0.194	-0.264	-0.072	-0.008	0.091	0.065	0.175	-0.064	-0.046	0.006
	P-value	0.442	0.243	0.109	0.668	0.960	0.598	0.699	0.294	0.701	0.784	0.970
Veillonellaceae_UCG_001	*r*	0.076	-0.005	0.114	-0.095	0.135	0.127	-0.270	0.224	-0.029	0.028	-0.238
	P-value	0.650	0.978	0.497	0.568	0.419	0.461	0.102	0.176	0.862	0.868	0.150
